# Experiences of Nursing Students Regarding Online Teaching and Learning During COVID‐19 Pandemic

**DOI:** 10.1155/nrp/8286447

**Published:** 2026-08-29

**Authors:** Keneilwe Bowane, Gofiwa Loeto, Bakang Bathoeng, Angelinah John, Tebogo Gorata Gaotlhobogwe, Olebile Clementine Bulang, Maripe-Perera Dorcas Basetsana, Kebope Mongie Kealeboga, Esther Seloilwe

**Affiliations:** ^1^ Gerald Clinic, Ministry of Health, Francistown, Botswana, saglik.gov.tr; ^2^ Lentsweletau, Ministry of Health, Kweneng, Botswana, sante.gov.ma; ^3^ Ntlhantlhe Clinic, Ministry of Health, Ntlhantlhe, Botswana, moh.koshi.gov.np; ^4^ Sir Seretse Kgama Baracks Hospital, Botswana Defence Force, Gaborone, Botswana; ^5^ Lotlhakane West Clinic, Ministry of Health, Lotlhakane, Botswana, moh.koshi.gov.np; ^6^ Sesung Health Post, Ministry of Health, Kweneng, Botswana, moh.koshi.gov.np; ^7^ University of Botswana, Gaborone, Botswana, ub.bw; ^8^ Retired, Gaborone, Botswana

**Keywords:** COVID-19, nursing education, online learning

## Abstract

**Background:**

Lockdowns during the COVID‐19 pandemic compelled universities to implement online teaching and learning methods to comply with social distancing measures. The University of Botswana transitioned from classroom face‐to‐face teaching to remote learning in response to the pandemic. This study aimed to explore the experiences of nursing students at the University of Botswana with online teaching and learning during the COVID‐19 outbreak. The results of the study have the potential to inform policies on how nursing education could respond to future pandemics and ensure continuity of learning.

**Design:**

The study utilised an explorative descriptive qualitative design to explore the students’ experiences using online teaching and learning activities during the COVID‐19 pandemic.

**Methods:**

Sixteen (16) students consented to participate in the study. Data were collected from September to November 2023 using a semistructured interview guide and analysed using content analysis. Trustworthiness was accomplished through credibility, dependability, confirmability and transferability. Further, the study used COREQ to ensure quality.

**Results:**

Five main themes and 14 subthemes were identified. The main themes are opportunities for online teaching and learning, students’ performances during the COVID‐19 pandemic, challenges of online teaching and learning, the need to improve online teaching and learning, and nursing as a profession and online learning.

**Conclusion:**

The study offered insights into how the COVID‐19 pandemic affected nursing students’ teaching and learning. Further research is recommended to guide nursing education and online learning policy.

**Clinical relevance**
• Online teaching and learning platforms are relevant and should be part of day‐to‐day clinical practice. It ensured continuity of care during the pandemic. In addition, it enhances the globalisation of nursing education by offering professional development to nurses in rural and metropolitan areas, allowing them to keep abreast of trends in nursing and e‐technology.

## 1. Introduction

In 2019, the world faced a deadly virus called COVID‐19, which originated in China [1]. COVID‐19 was later declared a pandemic by the World Health Organisation (WHO) when it became apparent that the virus had affected many people worldwide [2]. In response to mitigating the effects of COVID‐19, countries worldwide adopted measures such as border closures, curfews, travel permits, and lockdowns [3]. Educational institutions across the globe, in high‐, middle‐, and low‐resourced countries, were closed [4], and approximately 1.5 billion students from 191 countries were affected [5]. Such educational closures, lockdowns and movement restrictions compelled educational institutions to introduce online teaching and learning to protect both learners and educators from the disease and ensured that the education system processes continued amid the state of emergency. Hence, universities and educational institutions switched to or expanded to utilise online models and platforms for teaching and learning without any budget, as it was sudden [6–8].

Botswana schools, colleges, and universities had to adopt and adapt to the new systems to ensure that the mandate of academia was continued. Therefore, the University of Botswana (UB) adopted virtual learning opportunities and continued to blend teaching and learning methods which had always been available and used mainly by a few instructors and students [4, 9, 10]. For nursing students, online learning brought uncertainties, as traditionally, nursing is a contact profession that entails a lot of demonstration and close supervision from the nursing laboratories to the clinical area [7, 11–14]. However, COVID‐19 raged, and the School of Nursing at UB had no option but to implement the online lessons.

Online learning during COVID‐19 offered an opportunity for learning to continue without disruption [15]. Despite the implementation of social distancing and movement restrictions imposed during COVID‐19, students were allowed to attend classes from the comfort of their homes without exposing themselves to the virus and those who stayed off campus saved on transportation costs to and from school [4, 16]. However, the shift to online teaching and learning came with challenges. Most students missed classes due to network congestion and lacked the funds to purchase the necessary equipment to use for online learning, such as laptops and smartphones [17, 18]. Generally, most students found it challenging to adjust to technology and failed to concentrate, while for others, online education compromised the quality of training [19, 20]. Some students lacked the necessary resources to participate fully in online learning.

Despite the emergency, the students still had to fulfil their clinical hours, which is a requirement for the Nursing and Midwifery Council of Botswana. UB did not provide online clinical lab simulations; therefore, the school had to return the students to the clinical area for them to gain attitudinal and psychomotor skills. This experience presented fear, confusion and uncertainty for both students and lecturers in the School of Nursing, as COVID‐19 was unknown and had already left a deadly trail worldwide. A systematic review and meta‐synthesis of the experiences of students during the pandemic by Abbasi et al. [21] reported that students had uncertainties when they returned to the hospitals for their clinical practice. For instance, some students presented with psychological problems such as loneliness, fear, anxiety, and excessive worry of contracting the disease and infecting their loved ones at home. Other students felt disappointed to return to the clinical setting, feeling powerless and as though they were being thrown into danger.

On the other hand, other students were eager to return to the hospital setting, displaying resilience and happiness as they explored learning in the new, challenging situation presented by COVID‐19. Amidst the challenges faced by students during the clinical practice, there was nothing the School of Nursing could do, such as reducing the clinical hours, since the students had to meet certain hours to meet the requirements set by the Nursing and Midwifery Council of Botswana; hence, students had to go to the hospital for their clinical practice despite being faced with a challenging work environment. Furthermore, online teaching and learning require students to have high self‐discipline and self‐driven learning, which makes it difficult for lecturers to verify whether they are fully focused or merely passive [22]. The absence of in‐person meetings or communication between students and instructors, as well as among students themselves, contributed to feelings of isolation and low interest in classes [9, 20, 22–24]. To mitigate the challenges students experienced during online class presentations, UB offered mobile SIM packs with 1 GB of data. The innovations were adaptive and personalised, resulting in students enjoying and embracing new privileges such as flexible class participation, self‐paced learning, the cost‐effectiveness of online learning, internet access, and increased interactions with lecturers [17, 18]. However, online teaching and learning were a new change introduced for School of Nursing students at UB, and most students were not accustomed to it; hence, this study aimed to explore the experiences of UB nursing students with online teaching and learning during the COVID‐19 outbreak.

## 2. Design

The study used an explorative descriptive qualitative design. Qualitative studies explore how participants, given context, perceive and interpret a phenomenon [25]. The design was appropriate because it enabled the participants to describe their contextual experiences of online teaching and learning during the COVID‐19 pandemic.

## 3. Material and Methods

### 3.1. Participants

The study was conducted at UB School of Nursing on the main campus in the capital city of Gaborone. The School of Nursing at the UB has two cohorts of students: generic students (those admitted straight from high school with no nursing experience) and completion students (nurses with a diploma in nursing) (Bachelor of Nursing Science Curriculum, 2019). The study population consisted of all students, both generic and completion, at the 300 (Year 3) and 400 (Year 4) levels in the Bachelor of Nursing programme. There were a total of 168 students (64 at level 400) and 104 students at level 300. The participants were eligible for inclusion in the study only if they had experience with both traditional face‐to‐face teaching and online learning and had been placed in different health facilities for their clinical practice during the COVID‐19 pandemic. During that time, only students at levels 300 and 400 were eligible for inclusion in the study, since they had completed their clinical practice, and Years 1 and 2 had not yet started their clinical practice.

The researchers approached and purposively selected sixteen (16) participants who consented to participate. Purposive sampling helped the researchers to select participants who were knowledgeable and provided detailed, contextual information about the phenomenon [26, 27]. Furthermore, participants were asked whether they stayed on or off campus and whether they were at level 300 (Year 3) or 400 (Year 4) in their studies. The interviews were conducted from September to November 2023. Both genders were approached to reflect diversity. Face‐to‐face interviews were conducted with those who willingly consented to participate in the study. Interviews were conducted in the mornings between 1000 h and 1200 h, in the small nursing conference rooms to ensure privacy. The sample size was determined by data saturation and achieved by Participant 14. However, two more interviews were conducted to confirm further that no new information was generated. Hence, the study had a sample of 16 students.

### 3.2. Trustworthiness

Trustworthiness was assured through credibility, dependability, transferability and confirmability [28]. To ensure credibility, the researchers took time to engage with the participants. For example, researchers took the time to schedule appointments and to establish rapport and trust with participants. In addition, participants were allowed time to ask questions when they did not understand. After transcription and coding of the themes, the researcher also engaged with the participants to validate the themes. To ensure dependability, researchers listened to the recorded interviews and analysed them together using content analysis steps. Transferability was ensured by providing details on participants’ demographic data, the study setting, and the research methods used. In addition, debriefing exercises were conducted every day before and after data collection. Direct quotations were provided so that the reader may understand the views as expressed by the participants. To ensure confirmability, all researchers and supervisors were involved throughout the research process. Students conducted interviews, and study supervisors listened to them immediately and provided advice accordingly. Transcriptions were reviewed by supervisors and approved by participants for accuracy and relevance. In addition, the study was evaluated against the consolidated criteria for reporting qualitative research (COREQ) [29].

### 3.3. Data Collection

Following ethical clearance from the UB Institutional Review Board and permission from the School of Nursing, the researchers advertised the study throughout the university. Students were followed in their classrooms, and the study’s purpose was explained. Both on‐ and off‐campus students were encouraged to participate in the study. Prospective participants were invited to a meeting, where the researchers provided further details about the study. Those who were interested in participating in the study were asked to sign an informed consent form. An interview was conducted in a secure location to ensure privacy. Six (6) researchers (Keneilwe Bowane, Gofiwa Loeto, Bakang Bathoeng, Angelinah John, Tebogo Gorata Gaotlhobogwe and Olebile Clementine Bulang) were paired to interview participants. A semistructured interview guide consisting of four questions, developed from the literature on ‘COVID‐19 and nursing students’ online learning’, was used to collect data. The semistructured interview guide was developed using a thematic approach. It involved identifying research articles on ‘COVID‐19 and nursing students online learning’, perusing them for themes, generating codes, identifying emerging thematic meanings, reviewing them, and renaming them [30]. The guide was piloted with two participants who were not part of the study. A semistructured interview was preferred because it is exploratory and allows for probing and follow‐up questions [31]. In addition, two lecturers, Kebope Mongie Kealeboga and Dorcas Basetsana Maripe‐Perera, who are experienced in qualitative data analysis, supervised the research. Following data collection, the two supervisors listened to the audio recordings and advised as appropriate. Lastly, a highly experienced qualitative researcher, Professor Esther Seloilwe, ensured the quality of the process and that the three questions were maintained throughout the data collection.

The central questions were:

 What are the experiences of UB nursing students regarding online teaching and learning during the COVID‐19 pandemic?
 Which measures could be implemented to facilitate students’ teaching and learning during pandemics?
 What recommendations can be made to UB and the School of Nursing on measures that could enhance teaching and learning during pandemics?

Once the pilot study was completed, data were collected for 3 months from September to November 2023. The participants were assured that their information would be anonymised, and all participants consented to being recorded. To avoid bias, the researchers bracketed their views on the topic [32] by allowing participants to voice their knowledge and understanding regarding the topic without interruptions and avoiding the unconscious use of nonverbal body language, such as nodding the head to show understanding or agreement with what the participants were saying.

During the interview, the participants were encouraged to express themselves freely. Each interview took place in a secure place chosen by the participants for privacy. All interviews lasted 30–45 min and were recorded for data accuracy.

### 3.4. Data Analysis

Data were transcribed within 2 days of the interview and analysed using content analysis. Content analysis is a qualitative method that identifies, analyses, and interprets patterns within data [33]. The process involved transcribing and coding, where themes were organised and classified. Six members of the team (Keneilwe Bowane, Gofiwa Loeto, Bakang Bathoeng, Angelinah John, Tebogo Gorata Gaotlhobogwe and Olebile Clementine Bulang) transcribed the recordings. Two team members, Keneilwe Bowane and Gofiwa Loeto, coded the data, and the two supervisors, Kebope Mongie Kealeboga and Dorcas Basetsana Maripe‐Perera, confirmed the codes. Guidelines were provided to ensure consistency. Related codes were then categorised, and emerging themes were identified. Once the coding was complete, qualitative techniques were applied to interpret and analyse the coded data. Based on the analysis, conclusions were drawn, and the content was interpreted. Five themes and 14 subthemes emerged. Researchers related their findings to the research objectives, providing insights and implications. The study results were shared with students during a research seminar, though not all attended; the level 400 students had completed their training and exited the University.

### 3.5. Ethical Clearance

The UB Institutional Review Board granted permission for the study; reference UBR/RES/UNDERGRAD/BSN/002/2023. Students were followed in their classrooms and provided with details about the study. Those willing to participate in the study were given a consent form to sign. The following principles were accorded to participants: informed consent, autonomy, confidentiality and justice.

## 4. Results

### 4.1. Characteristics of the Participants

Sixteen (16) participants were interviewed for the study. The cohort comprised 7 (44%) females and 9 (56%) males, aged between 20 and 25 years, with a mean age of 22. All participants were single. Eight (8; 50%) were off‐campus students, and the other (8; 50%) were on‐campus students. Most students (10/16; 63%) were at level 400, and (6/16; 38%) were at level 300. Details are presented in Table 1.

**TABLE 1:** Demographic characteristics of participants.

Participants	Age	Gender	Marital status	Level of study	Accommodation status
P1	22	F	Single(s)	400	Off‐campus
P2	25	F	S	400	On‐campus
P3	22	F	S	400	Off‐campus
P4	21	F	S	400	Off‐campus
P5	21	M	S	300	On‐campus
P6	22	M	S	400	On‐campus
P7	22	M	S	400	Off‐campus
P8	23	F	S	400	On‐campus
P9	23	M	S	300	Off‐campus
P10	20	M	S	300	Off‐campus
P11	22	F	S	400	Off‐campus
P12	25	F	S	400	On‐campus
P13	23	M	S	400	On‐campus
P14	22	M	S	300	On‐campus
P15	21	M	S	300	Off‐campus
P16	25	M	S	300	On‐campus

*Note:* P‐participant, M‐male, F‐female, S‐single; mean age 22, male 9 and female 7; level 400 10, level 300 6, off‐campus 8 and on‐campus 8.

### 4.2. Synthesis of Findings

Five main themes and 14 subthemes were identified. The main themes are as follows: Opportunities of online teaching and learning, students’ performances during the COVID‐19 era, challenges of online teaching and learning, the need to improve online teaching and learning at the UB, and nursing as a profession and online learning. Details are presented in Table 2.

**TABLE 2:** Main themes and subtheme.

1. Opportunities of online teaching and learning	1.1 Access to materials and recordings 1.2 Reduced transport costs 1.3 Improved computer literacy

2. Students’ academic performance	2.1 Students’ good academic performance 2.2 Students’ poor academic performance

3. Challenges of online teaching and learning	3.1 Poor internet 3.2 Poor class attendance 3.3 Class disruptions (unmuted mics) 3.4 Failure to adapt to online teaching and learning 3.5 Faulty marking

4. The need to improve online teaching and learning in the University of Botswana	4.1 Increase internet connectivity and coverage 4.2 Educate learners and lecturers about online platforms 4.3 Improve monitoring systems for learners’ attendance

5. Nursing as a profession and online teaching and learning	5.1 Online teaching and learning is not suitable for a nursing course 5.2 Collaboration and teamwork

### 4.3. Theme 1: Opportunities of Online Teaching and Learning

Most participants found online teaching and learning to have opportunities. Some favoured online teaching and learning since lessons were recorded. They could access and revisit the recordings later, enhancing their comprehension of the material taught.

“*Some lecturers recorded their lessons, and we could revisit the content at our own time, and this enhanced understanding.”* (P3)

Some off‐campus participants reported that they did not have to travel to school to attend lessons, as is the case with face‐to‐face class teaching, which allowed them to save some money.

*“I saved a lot of money as I did not have to travel to school to attend lessons.”* (P9)

Other participants highlighted that online learning allowed them to explore different online platforms, such as Microsoft Teams, which they were unaware of before COVID‐19. The exposure then aided in improving their computer literacy and enhanced students’ learning.

“′*I learned about different online platforms that could be used in learning.”* (P1)

*“I became more computer literate because we used online platforms like Microsoft Teams and Zoom to conduct group meetings.”* (P6)

### 4.4. Theme 2: Students’ Academic Performance

COVID‐19 greatly affected the academic performance of the nursing students. For some, their academic grades excelled, while for others, the marks dropped. Those whose performance improved cited being able to revisit recorded course content, which allowed them to revise the material.

*“Online learning allowed me to revisit the class recordings. Unlike in a normal physical class, where sometimes it was difficult to hear my lecturer, and I had nowhere to refer. Hope you understand.”* (P)

However, some students indicated that their grades improved because they had opportunities to cheat during examinations. This is what one student said:

*“I excelled more during COVID-19 times because a lot of tests were not supervised, so it gave us an advantage we could copy.”* (P16)

Contributing factors to the poor performance were identified as follows: poor internet connection and failure to review/access lessons.

*“My results were not good… I missed a lot of content because of poor internet connection, and I could not even revisit the recorded lessons*.” (P14)

### 4.5. Theme 3: Challenges of Online Teaching and Learning

Teaching online was challenging. Some of the challenges included poor internet, poor class attendance, class disruptions and some students could not adapt to online teaching and learning.

Poor internet.

Nursing students, both on‐ and off‐campus, reported being unable to attend all online lessons due to poor internet connectivity and limited data.

*“I had difficulty with internet connectivity. My internet was poor, and I missed many lessons*.”(P2)

*“1 GB data provided was not adequate, it got finished whilst the lessons were on”* (P7)

Poor class attendance.

All students reported poor lesson attendance and cited reasons related to not having devices, poor network connectivity, and students being nuisances, as they were aware they were not being physically observed.

“Sometimes *I log on to my laptop during the lesson and continue with other things, for I know that the record will be available for review later”* (P 9)

*“I used a phone to access lessons, and where it failed over network or lack of data, I would miss the lesson”* (P11)

Class disruptions

The students also reported that class disruptions caused by unmuted microphones during lessons resulted in lost time and reduced learning. Some could not navigate through the technology.

“…*we were not aware of how to use Microsoft Teams, and one could unmute unknowingly and disturb the lesson.*” (P5)

Failure to adapt to online teaching and learning.

The students also reported that online teaching and learning were new to them. They also reported difficulty adapting to online teaching, as they were more accustomed to the traditional learning style.

“*I slept while attending online lessons, unlike when I was attending a physical lesson.”* (P6)

“*Umm, I could say I had a bad experience with online classes because I would sleep whilst attending the online lessons*.” (P4)

The student also reported that online teaching resulted in wrong grades being awarded due to a malfunction of the online marking system. They believed that they were wrong about the things they thought were correct.

*“Aaah, the marking system marked correct answers as wrong. I sometimes feel like I got wrong marks because of the system.”* (P12)

### 4.6. Theme 4: The Need to Improve Online Teaching and Learning at the UB

Online teaching and learning proved to be a resource during the COVID‐19 pandemic, though the shift came with some setbacks. Participants in the study, therefore, expressed the need for improvements in online teaching and learning across all aspects, including improving internet connectivity and speed and raising awareness among both lecturers and students, as these measures may improve the organisation of lessons and class attendance. One participant stated that

*“I think UB should improve the internet connectivity to avoid being cut off during the lesson; they should just increase the internet speed.”* (P16)

*“The UB management should educate lecturers on how to utilise online platforms so that they can operate them smoothly”* (P9)

Monitoring attendance was also one of the improvements that participants indicated could be done to ensure students do not miss lessons.

*“…to reduce cases of students not attending lessons, all the students should be required to introduce themselves at the beginning and the end of the lesson, even though it would be difficult to monitor”* (P7)

*“Lecturers could allocate marks for attendance”* (P15)

### 4.7. Theme 5: Nursing as a Profession and Online Teaching and Learning

Nursing curricula have always been designed to offer students both theoretical and clinical instruction to enhance the quality of training. Some participants argued that nursing is a practical course that requires hands‐on lessons for students to develop dexterity. These would not be guaranteed through online teaching and learning. This is what some participants stated:

*“Nursing is a practical course; students need to observe certain behaviours and attitudes on patients and practice in real-life situations.”* (P13)

“*Online teaching is good only in theory…we were not hands-on… we only watched videos which could not play at times”* (P8)

Some nursing students explained that online learning was not the best mode for them, as they needed to work in teams and collaborate with other healthcare team members, which was lacking in online teaching and learning.

“*We could no longer work in teams; we missed contact time with the lecturers, though some content really needed the presence of the lecturer to simplify”* (P11)

## 5. Discussion

The study explored the experiences of UB nursing students regarding online teaching and learning during the COVID‐19 outbreak. The findings revealed five main themes: opportunities for online teaching and learning; academic performance during the COVID‐19 era; challenges of online teaching; the need to improve online teaching and learning at the UB; and nursing as a profession and online teaching and learning, along with 14 subthemes. While some students found it challenging to adjust to online learning, others did so with ease.

Participants in this study reported several opportunities to teach and learn online, including reduced transportation costs, improved computer literacy, and access to recorded class materials, which students could access at their convenience, provided they had internet access. Other scholars observed similar findings [34, 35]. The use of technology in nursing education is unavoidable and has been associated with growth. Students’ assessment and class preparation rely on technology [4, 21, 36].

Excellent academic grades reported in this study were congruent with the existing literature, and these were linked to the flexibility of the method; students could attend lessons in the comfort of their homes, and online teaching and learning was convenient [4, 37–39].

On the other hand, the rise in grades could be due to cheating, as some participants in this study alluded to. During the pandemic, student supervision was inadequate because some assessments were conducted remotely, rendering traditional supervision methods ineffective for remote examinations [40]. In UB, assessments were taken remotely at home and in computer labs using online platforms, but there were no cameras to monitor them. In addition, some assessments were conducted in the computer labs; the ratio of invigilators to students was inadequate, thus creating a conducive environment for copying. A systematic review of the literature by Newton and Essex [41] on common forms of online examination cheating among university students indicated that academic misconduct was 29.9% pre–COVID‐19. However, during the COVID‐19 pandemic, it spiked to 54.7%, thereby threatening the reliability and validity of online testing. The reasons advanced for cheating included the perception that there was room for students to do so [41]. This could be true, since the UB lacks the advanced technology to administer and remotely monitor online examinations.

Such reports, therefore, call on the UB and other educational institutions to protect the integrity of their online assessment systems by ensuring that adequate resources are available to enhance the quality of examinations. Furthermore, these challenges arose from the sudden onset of the pandemic, which caught educational institutions unprepared and forced them to shift abruptly from traditional to online teaching and learning despite resource constraints [42].

The challenges of the online teaching and learning experience in the current study do not only occur in UB; students have reported them elsewhere. Several studies have reported that online teaching and learning during the COVID‐19 pandemic presented various challenges for teachers and students [23, 38, 43]. However, the challenges were particularly experienced in resource‐limited nations with insufficient infrastructure, where a large section of the population does not have access to the internet and electronic devices, and the speed is too low, indicating that not all students have access to high‐speed internet [4, 44]. As a result, students frequently struggled to access online classes and missed a lot of content, and thus the quality of the educational content was a concern, and the low attendance of lessons had a detrimental influence on their academic achievement [4, 20, 43, 45]. Scholars suggested that disturbances from unmuted microphones and student disengagement could be mitigated by instructors setting norms for online conduct and ensuring that all students have an equal opportunity to engage when they have questions or comments [46]. Participants reported that online systems constantly made errors in grading assessments. There is evidence suggesting that online marking systems are not without issues and are prone to technological errors such as incorrect recording and failure to preserve complete tests [39]. However, at UB, students have opportunities to have their marks validated or to address any examination‐related queries following assessments; hence, they cannot use these technical errors as a scapegoat for their failures.

Online teaching and learning must evolve to meet societal needs for improved, effective technology. The study results showed participants were dissatisfied with the strategies used and thus suggested that virtual technologies must be interactive, fostering collaboration and providing a supportive, conducive environment for learning. These demands by the participants have been documented in the literature, which further advocates that low‐ and middle‐income countries, such as Botswana, should use personalised technologies that are student‐centred, reflecting the learner preferences, content and cultural context where teaching and learning occur, as this will accord the learners the opportunity to modify the pace of their learning and be empowered to choose how and when they learn [4, 47–50].

While the UB planned to increase internet access and coverage by installing Wi‐Fi hotspots, expanding internet capacity, and enhancing network infrastructure [17], internet usage in Botswana stood at 1.12 million in January 2021. Usage increased by 2.0% between 2020 and 2021 [51]. By comparison, in 2020, an internet user spent 16 min, representing a 4% increase in a year [51]. At the dawn of 2025, there were 4.21 million active cellular mobile connections in Botswana, and many users had more than one mobile connection [51]. This picture depicted a positive step in the right direction for Botswana, while for an average Motswana, the struggle was massive. The current study highlighted the incompetence of some lecturers in using technology, resulting in their failure to utilise digital platforms in teaching, which scholars suggested could be mitigated through training in the form of workshops, online tutorials, and one‐on‐one sessions. Some literature suggests that technology must be personalised to individuals’ learning opportunities and capabilities and that the process must be based on age, attainment level, prior knowledge, and personal relevance. Learning, therefore, must be self‐paced, determined by the learner and guided by the learner’s preferences and cultural context [52–54]. Individual lecturers could access these services at their convenience.

Furthermore, participants in this study reported that online learning is unsuitable for nursing courses, as nursing requires a combination of practical and theoretical knowledge. Their views are supported by Haanes et al. [22] and Authement & Dormire [55], who believed that nurses exposed to a combination of theory and practice become qualified, gather practical experience, and are competent and successful. Studies have reported several hurdles to online nursing education. These include the difficulty of reproducing hands‐on learning experiences, retaining student interest, and encouraging effective communication, as it lacks nonverbal cues, teamwork, collaboration and networking with other healthcare professionals, such as physicians, pharmacists, and therapists. Students therefore missed the opportunity to appreciate the various roles of professionals, their responsibilities and accountability to patients and the essence of coordinated efforts in ensuring effective and efficient comprehensive care [22, 56]. Although evidence indicates online teaching is a valuable tool in nursing education, especially when conveying theoretical information, encouraging active learning and enhancing technology skills, it cannot substitute the necessity for in‐person clinical training and experience [12–14, 22, 55].

## 6. Lessons Learned

COVID‐19 challenged UB and the School of Nursing to implement their curriculum using methods beyond the face‐to‐face interaction typically used by nursing students. The UB had limited resources to support all students and lecturers, as the required changes were sudden. However, the participants in this study made the following recommendations: intensify supervision in exam rooms; upskill lecturers’ technical knowledge, as students know more than they do; and lock all unnecessary applications during the examination. Scholars are advocating for the proctoring of online assessments and examinations, in which automated systems will ensure fair, credible, monitored, and controlled assessments. The systems can detect and prevent cheating, enable real‐time remote assessment, monitor activity, restrict browsers, verify identity, recognise faces, perform audio analysis, and, through scanning, detect prohibited items such as mobile phones [57]. The identified materials are recorded in a real‐time database, stamped, and captured as snapshots to serve as proof [40, 58]. Some literature has suggested using cameras to record processes during online assessments and examinations [59, 60].

### 6.1. UB

The UB should increase internet connectivity coverage to reach all faculty blocks. In addition, there is a need to increase the internet packages allocated to students and improve lecturers’ computer literacy through continuous, recorded self‐learning computer classes to avoid disruptions during classes. Lastly, the UB should invest in advanced technological devices to better monitor students during tests and examinations, thereby increasing examination integrity as recommended by Arnautovski [61] and Noorbehbahani et al. [57].

### 6.2. Nursing Education

Lockdowns during COVID‐19 abruptly closed schools, leading to the loss of clinical hours for students. Nursing schools, government, and private organisations must collaborate to ensure that nursing education keeps pace with educational trends. Hence, the need to invest in eLearning technologies, such as virtual clinical simulations, that provide students with opportunities to continue practising while at home [62]. In addition, nursing schools should form partnerships with teaching hospitals to collaboratively design preceptorship programmes that enable senior, well‐experienced nurses to directly mentor and evaluate students during crises [62, 63]. Lastly, nursing education should update its curricula to include pandemic preparedness strategies and prioritise mental health for students and healthcare workers [64].

### 6.3. Nursing Research

Further research is needed to mitigate the impact of future pandemics, to explore the use of custom‐made, culturally appropriate e‐learning platforms to ensure continued education during pandemics, and to ensure platforms that will uphold the integrity, reliability, and validity of online teaching, learning, and assessments across the entire UB. These include studies that evaluate the quality and outcomes of simulation‐supported clinical learning and preceptor supervision of students [65].

## 7. Limitations of the Study

The study cannot be generalised to represent all the level 300 and 400 nursing students at UB. However, this study provided insight into how nursing students perceived online teaching and learning during the pandemic. A larger study that includes nursing students from other universities is recommended to better understand how the pandemic affected nursing education in Botswana. The findings of such a study could offer insight into how nursing education could structure its instructional methods to align its teaching with the unprecedented challenges presented by COVID‐19, which required curriculum implementation through online teaching and learning methods. The researchers acknowledge that the study used self‐reports during interviews, and as such, the quality of the collected data may be distorted.

## 8. Conclusion

The study reports on the experiences of students during the COVID‐19 pandemic and is centred on five main themes: Opportunities for online learning, students’ performance during the COVID‐19 pandemic, challenges of online teaching and learning, the need to improve online teaching and learning at the UB, and nursing as a profession and online learning. The participants recommended that universities invest in technology, as pandemics will strike whether planned or not; however, the effects could be lessened if proactive mitigation strategies to protect teaching and learning processes are in place. Therefore, the education sector in Botswana should ensure that health training institutions have active disaster preparedness plans.

## Funding

The study was not funded.

## Conflicts of Interest

The authors declare no conflicts of interest.

## Data Availability

Data can be availed upon reasonable request and approval by the relevant Institutional Review Board.
